# Liver Transplantation for Acute Liver Failure at 11-Week Gestation with Successful Maternal and Fetal Outcome

**DOI:** 10.1155/2012/484080

**Published:** 2012-11-26

**Authors:** Vinaya C. Maddukuri, Courtney D. Stephenson, Lon Eskind, William A. Ahrens, Preston Purdum, Mark W. Russo

**Affiliations:** ^1^Division of Hepatology, Carolinas Medical Center, 1000 Blythe Boulevard, Charlotte, NC 28203, USA; ^2^Department of Obstetrics and Gynecology, Carolinas Medical Center, 1000 Blythe Boulevard, Charlotte, NC 28203, USA; ^3^Department of Surgery, Carolinas Medical Center, 1000 Blythe Boulevard, Charlotte, NC 28203, USA; ^4^Department of Pathology, Carolinas Medical Center, 1000 Blythe Boulevard, Charlotte, NC 28203, USA; ^5^Carolinas Digestive Health Associates, Charlotte, NC 28211, USA; ^6^Transplant Center, Carolinas Medical Center, 3rd Floor Annex building, Charlotte, NC 28203, USA

## Abstract

Acute liver failure (ALF) during pregnancy is very uncommon. Pregnancy-specific liver conditions like hemolysis, elevated liver enzymes, and low platelets (HELLP) syndrome and acute fatty liver of pregnancy can cause ALF at term or postpartum, but, typically occur during the third trimester. Most of these patients recover spontaneously after delivery, but, on occasion, they require liver transplantation in the postpartum period. However, ALF during the first and second trimester of pregnancy requiring antepartum liver transplantation is rare. Only fifteen cases of liver transplantation during pregnancy have been reported, and very few occurred during the first trimester. We report a Woman who developed acute liver failure during the first trimester of pregnancy and underwent successful liver transplantation at 11-week gestation, followed by successful delivery of the fetus at 30 weeks. To our knowledge, this is the earliest case of successful liver transplantation during pregnancy followed by successful fetal outcome. We discuss management of the patient and fetus before, during, and after liver transplantation and review the literature on antepartum liver transplant in pregnancy.

## 1. Introduction

Abnormal liver tests can be seen in 3% to 5% of pregnant women and are usually due to pregnancy related conditions [1]. Pregnancy related conditions, such as intrahepatic cholestasis of pregnancy (ICP), pre-eclampsia, HELLP (hemolysis, elevated liver enzymes, and low platelets) syndrome, and acute fatty liver of pregnancy (AFLP) [1], almost always occur during the third trimester. Conditions unrelated to pregnancy, such as viral hepatitis, gallstones, and drug-induced liver injury can occur at any time during pregnancy, including the first and second trimesters, and cause significant liver dysfunction. However, acute liver failure (ALF) during pregnancy is very rare, and the exact incidence is unknown. HELLP syndrome and AFLP can cause ALF late in pregnancy or postpartum, and most patients recover spontaneously after delivery [2]. On occasion, these patients may require orthotopic liver transplantation (OLT) in the immediate postpartum period [2]. According to the European Liver Transplantation Registry (ELTR), ten of 75,530 liver transplants performed between 1968 and 2008 were performed for acute liver failure due to AFLP and HELLP syndrome [3]. 

Liver transplant during pregnancy (antepartum or before delivery) has been previously reported (Table 2). However, OLT during the first trimester has been reported in only two previous cases, of which, only one case resulted in successful delivery of the fetus. We report a 29 year-old woman who suffered cryptogenic ALF in the 11th week of her pregnancy requiring emergent antepartum OLT. She continued her pregnancy after transplant and delivered at 30 weeks, with a successful maternal and fetal outcome. Both mother and child are healthy 4 years after liver transplantation and delivery. 

## 2. Case

A 29 year-old woman, G1 P0, at week 10 of her pregnancy, was admitted with five days of jaundice, nausea, diarrhea, and right upper quadrant pain. She otherwise had no significant past medical history and was not taking any medications, including over the counter medications, except for prenatal vitamins. There was no family history of liver disease. On admission, she was jaundiced, and her abdomen was appropriate for her gestational age with no evidence of ascites. She had no asterixis or encephalopathy on presentation. Admission laboratory tests revealed acute hepatitis and were as follows (Table 1): total bilirubin 16.4 mg/dL, aspartate aminotransferase (AST) 1001 IU/L, alanine aminotransferase (ALT) 1269 IU/L, alkaline phosphatase 105 IU/L, gamma glutamyl transferase (GGT) 66 IU/L, serum albumin 2.2 gm/dL, serum creatinin 0.6 mg/dL, white blood cell count (WBC) 12.6 K/*μ*L, platelets 161 K/*μ*L, and hemoglobin 12.6 gm/dL. 

A thorough evaluation for the etiology of acute hepatitis was unrevealed. The following tests were negative: hepatitis A IgM, hepatitis B surface antigen, hepatitis B core antibody [total & IgM], hepatitis C antibody, hepatitis E antibody, cytomegalovirus (CMV) IgM, Epstein-Barr virus IgM, human immunodeficiency virus antibodies, herpes simplex virus (HSV) IgM, varicella IgM, antinuclear antibodies, antimitochondrial antibodies, and antismooth muscle antibodies. The serum ceruloplasmin and immunoglobulin levels [IgG, IgM, and IgA] and plasma alpha −1 antitrypsin level were within the normal range. The serum acetaminophen level was <10 mcg/mL, and urine drug screen was negative. An abdominal ultrasound with Dopplers showed heterogeneous appearing liver with patent hepatic vasculature, without intra- or extrahepatic biliary dilatation. An MRI of the abdomen showed heterogeneous enhancement of the liver with periportal edema, patent portal, and hepatic veins but no cirrhosis. 

**Figure 1:**
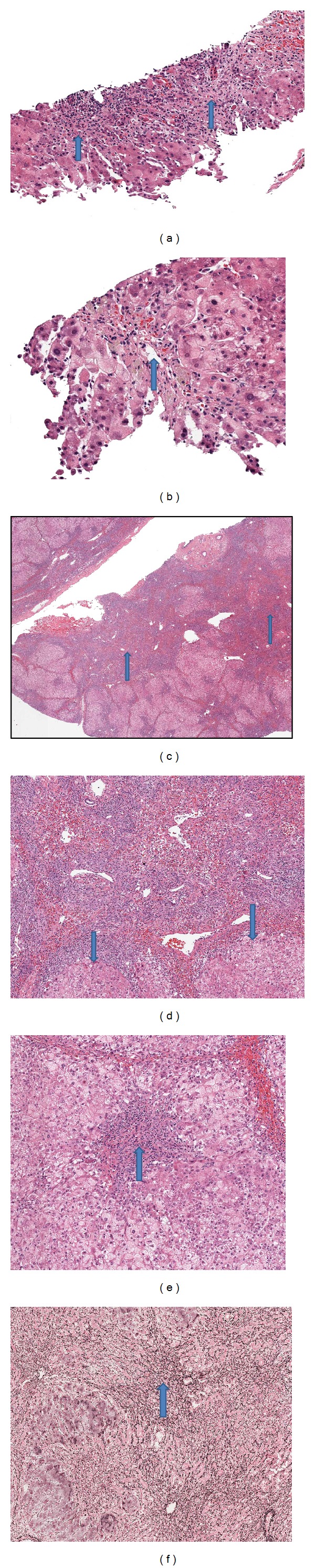
(a) Transjugular biopsy-portal tract showing inflammation with bile ductular proliferation and interface hepatitis (arrows) (H&E 200x). (b) Transjugular biopsy-central vein (arrow) with pericentral hepatocyte dropout, ballooning degeneration, and hemorrhage (H&E 200x). (c) Explant showing architectural collapse (arrows) (H&E 40x). (d) Explant with interface between area of collapse and residual viable parenchyma (arrows) (H&E 100x). (e) Explant with portal/interface hepatitis (arrow). Periportal hepatocytes showing ballooning/cytoplasmic clearing (H&E 100x). (f) Reticulin stain highlights areas of collapse (arrow) adjacent to viable parenchyma.

On the third hospital day, a transjugular liver biopsy was performed that showed intense, active, interface hepatitis (Figures 1(a) and 1(b)) with lymphocytes, neutrophils, and scant plasma cells, bile ductular proliferation, and massive hepatocyte dropout. A Masson's trichrome stain showed bridging fibrosis (stage 3) with lobular architectural collapse. Liver biopsy HSV stains were negative. She received intravenous methylprednisolone for presumed autoimmune hepatitis (AIH), but her condition continued to worsen. She underwent emergent evaluation for liver transplantation and was listed for liver transplant.

On day five of admission, she developed intermittent vaginal bleeding because of coagulopathy. Her Factor V and Factor VII activity levels on day seven of admission were very low at 6% and 4%, respectively. On the morning of the eighth day, she was lethargic and developed asterixis, but was arousable, consistent with stage 3 hepatic encephalopathy. On the eight hospital day, she underwent orthotopic liver transplantation from a 25 year-old donor who died from a self-inflicted gunshot wound to the head. The donor was CMV positive, and the patient was CMV negative.

Abdominal transplant surgery, hepatology, high-risk obstetrics, and anesthesiology teams were involved in her care throughout the hospitalization. Pretransplant obstetric ultrasound was performed by high-risk obstetrics team to confirm fetal viability and to accurately date the pregnancy. The potential complications of posttransplant immunosuppression on the pregnancy were discussed with the patient, by the obstetrics team, before transplant. Medical termination of pregnancy was not recommended as there was no evidence to suggest that pregnancy caused or worsened the patient's liver disease. 

Liver transplantation was performed with venovenous bypass. A suprahepatic caval anastomosis was created between the donor liver and the hepatic vein confluence of the recipient. A common cuff of gastroduodenal artery and proper hepatic artery was fashioned on the recipient's side and using the celiac axis of the donor, an end-to-end hepatic arterial anastomosis was created. An end-to-end portal venous anastomosis and an end-to-end choledochochledochostomy were performed. There was excellent perfusion of the liver, and it synthesized bile within 5 minutes of recirculation. She remained hemodynamically stable during the procedure without any vasopressor requirement. She received one unit of packed red blood cells, nine units of fresh frozen plasma and ten units of platelets during surgery. A posttransplant X-ray was not obtained due to her pregnancy. Intraoperative fetal monitoring was not performed during the liver transplant since the fetus was less than 24 weeks and not viable. Postoperatively, an obstetric ultrasound was performed which confirmed fetal cardiac activity and normal amniotic fluid volume.

 Pathology from her explanted liver revealed a small organ (total weight 509 grams), prominent interface hepatitis, extensive hepatocyte necrosis, and confluent collapse (Figures 1(c)–1(f)), but there was focal preservation of some lobules (Figure 1(d)). Interestingly, Masson's trichrome stain of the explanted liver showed very little fibrosis. The findings from explant were different from the transjugular liver biopsy which showed stage 3 fibrosis. 

Tacrolimus was administered with goal plasma trough levels of 10–12 ng/mL, and our center's standard steroid protocol were administered for immunosuppression (Table 2) after liver transplantation. Although mycophenolate mofetil is part of our center's immunosuppression protocol for patients transplanted for ALF, it was avoided due to its potential teratogenic effects. CMV prophylaxis was initiated with intravenous ganciclovir in the immediate post-operative period, and it was changed to oral valganciclovir at the time of discharge. Trimethoprim-sulfamethoxazole for pneumocystis prophylaxis was started after the first trimester. She did not have any immediate posttransplant complications and was discharged on hospital day 16, seven days after surgery, with a viable intrauterine pregnancy. 

She was closely followed by abdominal transplant surgery, hepatology and high-risk obstetrics teams after discharge. Outpatient laboratory testing and imaging studies were performed per respective team protocols. Specifically, obstetric ultrasounds were performed every 2-3 weeks to monitor fetal well-being. The graft function remained stable, and her pregnancy progressed without any complications until 24 weeks. At 24 weeks, she developed severe, diffuse pruritus due to intrahepatic cholestasis of pregnancy (ICP). Blood work revealed elevated alkaline phosphatase and gamma glutamyl transferase (Table 1). Her serum bile acid levels were also elevated at 44 *μ*mol/L, but total bilirubin and aminotransferases were normal. There was no evidence of liver graft rejection or posttransplant biliary stricture. She was treated with ursodeoxycholic acid (UDCA) and cholestyramine for the relief of pruritus. She also developed gestational diabetes at 24 weeks and was treated appropriately with insulin. After the diagnosis of ICP, she was evaluated weekly for fetal well-being by ultrasound biophysical profile studies. At 30 weeks gestation, fetal growth began to slow, and the amniotic fluid volume started decreasing. At that point, she received betamethasone for fetal lung maturation. During the same week, she experienced spontaneous rupture of membranes and delivered by normal vaginal delivery. She had no postpartum complications. The newborn was a healthy female weighing 3 lbs, 12 ounces (1700 grams) with an Apgar score of 9 and 9 at one and five minutes, respectively. The child did not breast feed. She is now four years old, healthy, and reaching her age with appropriate developmental milestones.

## 3. Discussion

Although planned pregnancy after liver transplantation is relatively common, acute liver failure requiring liver transplantation during pregnancy is rare. Liver transplantation during the first trimester of pregnancy is an exceptionally uncommon event. A search in the literature found only fifteen cases of antepartum liver transplantation, and only six (40%) of those cases resulted in successful delivery of the fetus (Table 2). Among those six cases, first trimester (14 weeks or less) liver transplantation was performed only in two patients. Spontaneous abortion occurred in one of those two, and the other pregnancy resulted in a successful fetal outcome. Thus, our case represents only the second case of successful liver transplantation during the first trimester, followed by a successful pregnancy outcome, reported in the literature. To our knowledge, our case is the earliest successful liver transplant during pregnancy with successful fetal outcome. Furthermore, we provide long term followup of the child who has met developmental milestones.

 Management of ALF in pregnancy is complicated as both maternal and fetal well-being need to be considered, although maternal outcome takes precedent. An algorithm proposed by Greenberg et al. in 2009 is a useful tool in the management of ALF in pregnancy [4]. 

We used a combination of corticosteroids and tacrolimus for posttransplant immunosuppression. Corticosteroids and tacrolimus are categorized by Food and Drug Administration (FDA) as pregnancy category B and C medications, respectively [5]. We choose tacrolimus instead of cyclosporine as it is a more potent calcineurin inhibitor, with lower rates of maternal complications like renal dysfunction, pre-eclampsia, and hypertension [5]. We avoided mycophenolate mofetil because it is teratogenic, and (FDA category D) and is associated with very high rates of first trimester pregnancy loss and birth defects [5]. In one report on 33 pregnancies exposed to mycophenolate mofetil, (from National Transplant Pregnancy Registry (NTPR)), 15 (45%) pregnancies resulted in spontaneous abortions and 4 among 18 live births (22%) had congenital malformations [5]. Azathioprine is also FDA category D medication [3] and should be avoided. Other immunosuppressive agents such as muromonab, antithymocyte globulin, and sirolimus belong to FDA category C, and available data on safety in pregnancy is insufficient [3]. Among the fifteen case reports that we reviewed, information about immunosuppression was provided in eleven reports (Table 2). In six of these eleven patients (54%), similar to our case, a combination of corticosteroids and tacrolimus was used [6–11]. Our patient received trimethoprim-sulfamethoxazole for pneumocystis prophylaxis. It was started after the first trimester as the medication is a folate antagonist (pregnancy category C), with potential for teratogenicity [12]. We initiated intravenous ganciclovir (Cytovene) immediately after transplant for CMV prophylaxis and later changed to oral valganciclovir (Valcyte) at the time of discharge. Both ganciclovir and valganciclovir are pregnancy category C medications (Cytovene and Valcyte prescribing information. Roche Laboratories, Inc., 2010), but the benefits of treatment outweighed the risks in our patient.

 Etiologies of acute liver failure during pregnancy include causes that occur in the general population or pregnancy specific conditions, such as HELLP syndrome and AFLP [2]. In many cases a cause is not determined. In a study from India, a workup for an etiology of ALF was negative in 68 of 249 (27.9%) of pregnant patients with ALF [13]. In the fifteen cases that we reviewed, six (40%) were cryptogenic [7–9, 14–16], three (20%) were due to hepatitis B [17–19], two (13%) were due to drug induced liver injury [11, 20], two (13%) were due to AIH [6, 21], one (6%) patient had PBC [10], and a cause was not reported in one case [22] (Table 2). It is interesting that none of the fifteen patients that we reviewed developed ALF due to HELLP syndrome, pre-eclampsia or AFLP. In our patient, a thorough evaluation for a cause of ALF, including the histology of explanted liver, did not reveal a definitive cause. Autoimmune hepatitis was suspected based upon both transjugular liver biopsy and explant histology; however, her autoimmune serologies were negative, and she did not respond to corticosteroids. 

Unfavorable fetal outcomes were noted in nine of the fifteen cases (60%) that we reviewed (Table 2). Four fetal deaths (26%) [6, 16, 19, 22], two neonatal deaths (13%) [17, 20], two spontaneous abortions (13%) [8, 15], and one artificial abortion (6%) [7] were reported. Favorable fetal outcomes were noted in six of the fifteen cases (40%). Three neonates were born prematurely [9, 18, 21], and the remaining three [10, 11, 14] were born at term. Fetal malformation rate in pregnant women treated with corticosteroids is 4%, and tacrolimus is 6% which is slightly higher than the malformation rate of 2-3% in general population [5]. Fortunately, no congenital malformations were present in our case. Corticosteroids are associated with premature rupture of membranes [5]. Our patient developed premature rupture of membranes at 30 weeks and delivered a premature, low-birth weight (1700 grams) baby. Neonatal adrenal insufficiency and infections may occur in corticosteroid-treated pregnancies [5], but those complications were not seen in our case. The baby survived without any complications in spite of prematurity. The child is now 4 years old, and she has met all her developmental milestones with no cognitive or physical deficits.

 Other lessons that can be learned from our case include preoperative management of the pregnant patient with acute liver failure. Preoperatively, we did not routinely give fresh frozen plasma to prevent uterine bleeding for her coagulopathy but managed her similar to nonpregnant patients. She was transfused with fresh frozen plasma, as needed, in an attempt to control her intermittent vaginal bleeding that occurred three days before her liver transplantation. She received fresh frozen plasma for procedures, such as transjugular liver biopsy. Since the fetus had not reached viability (24 weeks) at the time of transplant, pre- and intraoperative fetal monitoring were not conducted as that would not have altered the management.

An interesting and particularly concerning event was the development of intrahepatic cholestasis of pregnancy (ICP) at 24-week gestation. In general, ICP is a self-limited condition which resolves after delivery of the fetus and usually occurs in the second half of pregnancy [1]. Fetal and perinatal mortality associated with ICP is 0.4%−1.4%, but there is no increased maternal mortality [1]. The goal of the treatment is to control maternal pruritus and prevent fetal mortality. Our patient was treated with UDCA and cholestyramine for symptom relief, and ultrasound biophysical profile studies were performed weekly to document fetal well-being. 

In conclusion, acute liver failure during pregnancy requiring liver transplantation can be performed with successful fetal outcome, even during the first trimester of pregnancy. Intraoperative management did not include fetal monitoring, but high-risk obstetrics was present in the operating room at the time of transplant. Hemodynamic monitoring and volume management were performed similar to nonpregnant patients undergoing liver transplantation. A multidisciplinary approach with hepatology, transplant surgery, high-risk obstetrics, and anesthesiology may result in a healthy mother and child.

## Figures and Tables

**Table 1:** Selected laboratory values.

Day→	Day 1 (presentation)	2	3	4	5	6	7	8 transplant	9	10	16 discharge	Week 24	Week 26
Lab↓													
Creatinine (mg/dL)	0.6	0.7	0.6	0.7	0.7	0.7	0.6	0.7	0.7	1.0	0.6	1.0	1.1
Albumin (g/dL)	2.2	2.6	2.2	2.4	2.4	2.3	2.3	2.5	2.6	2.9	2.5	3.1	3.1
Total bilirubin (mg/dL)	16.4	16.2	16.0	19.0	21.2	22.1	22.3	24.0	13	4.4	2.6	0.8	0.5
Alkaline phosphatase (IU/L)	105	116	112	143	152	176	166	172	98	63	56	152	152
AST (IU/L)	1001	757	684	729	733	637	580	567	808	370	38	29	30
ALT (IU/L)	1269	883	818	842	834	726	633	607	582	357	117	53	50
INR (s)	3.3	1.7*	2.6	2.9	3.2	3.5	4	5	1.4	1.2			
GGT (IU/L)	66								37	24	50	119	131
Bile acids (*μ*mol/L)												44	

**Table 2:** Review of cases of liver transplantation during pregnancy.

Reference	Diagnosis	Gestational age at transplant	Immunosuppression	Outcome
Morris et al. [20]	Acute liver failure from drug induced liver injury	27 weeks	Prednisone and cyclosporine	Successful deceased donor liver transplant and neonatal death
Laifer et al. [6, 17]	Fulminant Hepatitis B	26 weeks	Prednisone and cyclosporine	Delivery by c section at 28 weeks and neonatal death
Fair et al. [18]	21 y/o woman with acute liver failure from Hepatitis B	22 weeks gestation	Steroids, OKT3, and cyclosporine	Deceased donor liver transplant with primary malfunction, followed by successful retransplantation on postop day 2. C section at 30 weeks. Fetus survived and healthy. Fetus had IUGR and severe oligohydramnios
Moreno et al. [14]	32 y/o female with cryptogenic acute liver failure	27 weeks	Methylprednisolone, cyclosporine, and azathioprine	Successful deceased donor liver transplantation Fetus survived. C-section at 39 weeks. Infant healthy at 11 months
Hamilton et al. [19]	20 y/o woman with acute liver failure from Hepatitis B	18 weeks	n/a	Successful deceased donor liver transplantation. Fetal death on posttransplant day 5
Finlay et al. [22]	Acute liver failure	17 weeks	n/a	Successful deceased donor liver transplantation. Fetal death at 22 weeks
Lo et al. [15]	25 y/o with cryptogenic acute liver failure	26 weeks	n/a	Successful left lobe living donor liver transplantation. Spontaneous abortion posttransplant day 2
Catnach et al. [21]	27 y/o woman with end stage autoimmune hepatitis	20 weeks	Cyclosporine, prednisolone, and azathioprine	Successful deceased donor liver transplantation. Successful delivery at 28 weeks. Recipient developed CMV infection
Laifer et al. [6, 17]	30 y/o female decompensated cirrhosis from autoimmune liver disease	23 weeks	Tacrolimus and prednisone	Fetal death posttransplant day 6
Eguchi et al. [7]	28 y/o woman with cryptogenic acute liver failure	15 weeks	Prednisone and tacrolimus	Successful adult living donor liver transplant. Artificial abortion posttransplant day 31. Recipient developed CMV infection
Kato et al. [8]	31 y/o female with cryptogenic acute liver failure	14 weeks	Methylprednisolone, tacrolimus, and 2 days of mycophenolate mofetil	Left lateral segment adult to adult living donor liver transplantation. Spontaneous abortion immediately after transplant
Jarufe et al. [9]	35 y/o female with cryptogenic acute liver failure	22 weeks	Prednisone and tacrolimus	Successful deceased donor liver transplantation. Delivery at 27 weeks of gestation. Normal psychomotor development at 1-year followup
Jankovic et al. [10]	26 y/o female with cryptogenic familial biliary cirrhosis	13.5 weeks	Tacrolimus, azathioprine, and prednisolone	Successful liver transplantation. Vaginal delivery at 36 weeks
Anders et al. [16]	18 y/o female with cryptogenic acute liver failure	20 weeks	n/a	Successful liver transplantation. Fetal death 48 hours after transplantation
Sequeira et al. [11]	36 y/o female with drug induced acute liver failure due to propylthiouracil	18 weeks	Tacrolimus, cyclosporine, and prednisolone	Successful liver transplantation. Elective cesarean section at 37 weeks. Child had IUGR, oligohydramnios, decreased cerebral volume, ventriculomegaly, microcephaly, seizures, and delayed developmental milestones
Current case	29 y/o female with cryptogenic acute liver failure	11 weeks	Tacrolimus and prednisone	Successful deceased donor liver transplantation. Healthy female fetus delivered at 30 weeks. Normal psychomotor development at 4-year followup
